# High temperature measurements and condensed matter analysis of the thermo-physical properties of ThO_2_

**DOI:** 10.1038/s41598-018-21406-w

**Published:** 2018-03-22

**Authors:** T. R. Pavlov, T. Wangle, M. R. Wenman, V. Tyrpekl, L. Vlahovic, D. Robba, P. Van Uffelen, R. J. M. Konings, R. W. Grimes

**Affiliations:** 1 European Commission, Joint Research Centre, Institute for Transuranium Elements, P.O. Box 2340, 76125 Karlsruhe, Germany; 2 Department of Materials and Centre for Nuclear Engineering, Imperial College London, Royal School of Mines, London, SW7 2AZ UK; 3 SCK•CEN, Institute of Nuclear Materials Science, Boeretang 200, 2400 Mol, Belgium; 4 Department of Materials Engineering, KU Leuven, Kasteelpark Arenberg 44, 3001 Heverlee, Belgium

## Abstract

Values are presented for thermal conductivity, specific heat, spectral and total hemispherical emissivity of ThO_2_ (a potential nuclear fuel material) in a temperature range representative of a nuclear accident - 2000 K to 3050 K. For the first time direct measurements of thermal conductivity have been carried out on ThO_2_ at such high temperatures, clearly showing the property does not decrease above 2000 K. This could be understood in terms of an electronic contribution (arising from defect induced donor/acceptor states) compensating the degradation of lattice thermal conductivity. The increase in total hemispherical emissivity and visible/near-infrared spectral emissivity is consistent with the formation of donor/acceptor states in the band gap of ThO_2_. The electronic population of these defect states increases with temperature and hence more incoming photons (in the visible and near-infrared wavelength range) can be absorbed. A solid state physics model is used to interpret the experimental results. Specific heat and thermal expansion coefficient increase at high temperatures due to the formation of defects, in particular oxygen Frenkel pairs. Prior to melting a gradual increase to a maximum value is predicted in both properties. These maxima mark the onset of saturation of oxygen interstitial sites.

## Introduction

Thoria is a possible replacement nuclear fuel material for urania. There are a number of reasons for investigating, designing and implementing a thorium fuel cycle. Thorium is 3–4 times more abundant than uranium^1^. There is some evidence suggesting that ThO_2_ can better retain fission products at lower burnups^2,3^. Thorium possesses a good breeding efficiency over a wider neutron energy spectrum compared to uranium and thorium can be bred more efficiently via thermal neutrons^4^. Thoria is considered more proliferation resistant compared to urania, partly due to highly active decay products formed early on during irradiation, which require special handling facilities but noting that proliferation risks still exist^5^ via the extraction of Pa^233^ (early-on during irradiation) and its subsequent decay to U^233^. Finally, ThO_2_ exhibits superior thermo-physical properties such as a higher melting point compared to UO_2_^6^, as well as higher thermal conductivity up to 2000 K^7,8^. Various authors investigated the properties of ThO_2_ up to 2000 K. There is, however, a lack of experimental data at higher temperatures for the thermo-physical properties of ThO_2_, which is an impediment to predictions of thoria fuel performance under accident conditions.

At temperatures up to 2000 K specific heat has been measured by a significant number of authors^9,10^, while above 2000 K few studies exist. Amongst these few works are the experimental investigations of Ronchi *et al*.^11^ and Ralph *et al*.^12^ Their work has provided evidence for a significant increase in this property above 2000 K, most likely due to the formation of oxygen defects. Furthermore, Ronchi *et al*. measured a steep increase in specific heat followed by a decrease, marking a maximum at around 3100 K. Thermal conductivity has also been investigated by various authors at temperatures up to 2000 K^13,14^. At higher temperatures, Weilbacher^15^ and Faucher^16^ have measured thermal diffusivity and from the density and specific heat one could estimate the thermal conductivity. However, as previously discussed the high temperature specific heat values are scarce and differ significantly. This introduces a degree of uncertainty when calculating thermal conductivity based on an indirect approach by using literature values for specific heat and density. Thus, the aim of this work is to establish experimental values of thermal conductivity, and specific heat in the temperature range 2000 K to 3050 K via a semi-direct laser flash method^17,18^. It is described as semi-direct, because specific heat and thermal conductivity are determined simultaneously, however the method requires density as input. The method and apparatus have been also used to determine thermal diffusivity, spectral emissivity and total hemispherical emissivity of ThO_2_, in the temperature range 2000 K to 3050 K. Furthermore, the temperature dependence of the experimental results will be described using a solid state physics model^18^, which is based on the governing physical processes.

## Methodology

### Sample preparation

Green pellets were prepared according to the process proposed by Wangle *et al*.^19^. A solution of 1 mol/l Th(NO_3_)_4_ in HNO_3_ (Solvay) was added to a 0.75 mol/l oxalic acid (Sigma-Aldrich) solution (5% excess), which was cooled to 10 °C. After precipitation, the solution was digested for 15 minutes, filtered and dried at 70 °C overnight. The filter cake was finally forced through a 300 µm sieve. The sieved Th(C_2_O_4_)_2_∙*n*H_2_O precursor was calcined to ThO_2_ in a Nabertherm LT 9/13/P330 muffle furnace at 1 °C/min at 350 °C and held for 12 h, then heated at 1 °C/min to 700 °C and held for 1 h. The powder was loaded without further manipulation into a stearic acid coated 6.9 mm diameter steel die. The green pellets were pressed in a uniaxial Atlas Auto 8T press at 300 MPa. Sintering was performed in a Linn HT 1800 Moly high temperature furnace, by heating to 1700 °C at 5 °C/min with a dwell time of 8 h. An atmosphere of Ar + 5% H_2_ with a small admixture of Ar + 0.5% O_2_ was used to give an oxygen potential of −420 kJ/mol at 1700 °C.

### Spectral emissivity

Two independent methods for emissivity measurement were employed – a ratio pyrometer (RP) method and spectropyrometer (SP) method. In the case of the SP method, a spectropyrometer measures the radiance on the front side at 256 wavelengths in the range 500 nm to 1000 nm. Assuming constant emissivity in the range 590 nm to 700 nm, the spectral emissivity is obtained from a linear fit of inverse radiance temperature vs. wavelength. This method has been described in detail previously^17^.

The RP method utilizes a ratio pyrometer (two color pyrometer), which measures radiances at two different wavelengths. The measured radiances can be related using Planck’s law. The problem can be further simplified using two assumptions: (1) Wien’s approximation; (2) constant emissivity due to the relative proximity between *λ*_1_ and *λ*_2_. The resulting equation is used to determine the black body temperature via two-color or ratio pyrometry:

1 $$\frac{L({T}_{R},{\lambda }_{1})}{L({T}_{R},{\lambda }_{2})}={(\frac{{\lambda }_{2}}{{\lambda }_{1}})}^{5}({e}^{\{\frac{{c}_{2}}{{T}_{B}}(\frac{1}{{\lambda }_{2}}-\frac{1}{{\lambda }_{1}})\}})$$

where *L* is the radiance (W m^−3^ s r^−1^), *λ*_1_ is the first wavelength (m), *λ*_2_ is the second wavelength (m), *T*_*R*_ is the radiance temperature (K), *T*_*B*_ is the black- body temperature (K) and *c*_2_ is the second radiation constant (m K). From the ratio of the measured radiances at the two wavelengths the black-body temperature can be calculated. Simultaneously the radiance temperature at 645 nm is measured via another single wavelength pyrometer. Hence, using the measured black body and radiance temperatures and by employing Wien’s law the spectral emissivity at 645 nm is retrieved.

### Laser flash

**Figure 1:**
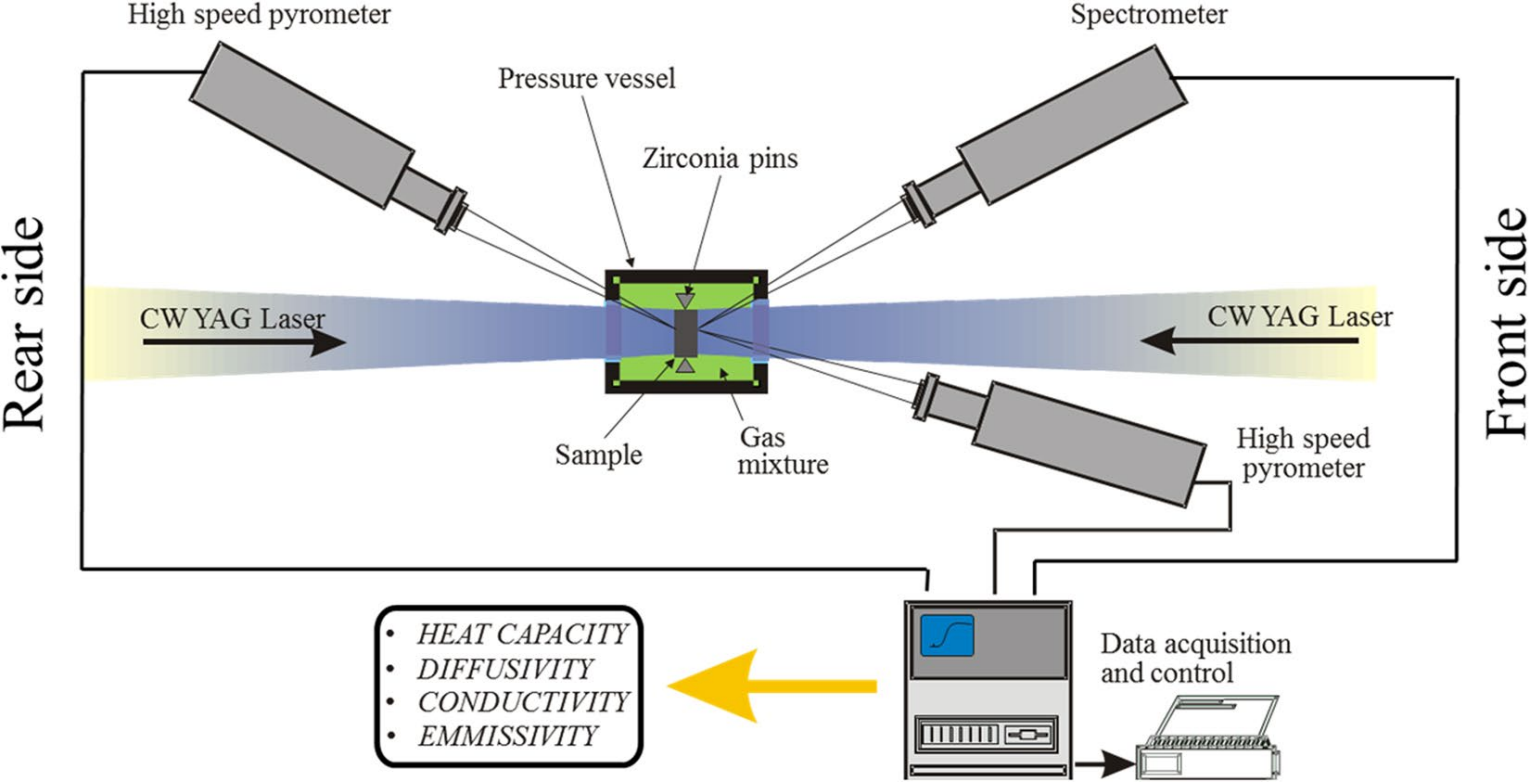
Schematic of laser flash experimental set-up^17^.

The experiment is based on the laser flash set-up displayed in Fig. 1, with parameters reported in Table 1. The ThO_2_ samples are held by three zirconia pins, attached to a graphite sample holder. These are placed in a pressure vessel under a pure oxygen atmosphere (purity of ≈99.99%) at 3 bar. The transparent sapphire windows of the chamber allow for two continuous wavelength (Nd-YAG with λ = 1064 nm) lasers to preheat the front and rear side of the sample. Due to the semi-transparent nature of ThO_2_ a tailored preheating procedure is implemented. First of all, the rear laser is turned on, in order to continuously irradiate the sample surface. As the material is transparent to the laser’s light, no significant temperature increase is recorded. Subsequently the front laser is turned on and its power is manually increased to a critical power (or temperature), at which point the sample begins to absorb the laser’s light much more readily and the temperature rises. The process of increasing radiative absorption is extremely prompt and necessitates the immediate switch-off of the front laser, in order to avoid sample melting. At this point the rear laser is still heating the sample, maintaining it at a lower temperature (1500 K to 1700 K), which is high enough for the sample to remain absorptive to the laser’s radiation. At this stage the sample temperature is stable and controllable. The equilibrium temperature is then varied between 2000 K and 3100 K.

**Table 1:** Summary of ThO_2_ sample characteristics and laser beam parameters.

Parameter (units)	Value range
room temperature density ρ_298_ (kg m^−3^)	9300
porosity (%)	7
thickness (mm)	1.56
radius (mm)	2.825
beam spot radius (mm)	1.5
pulse duration (ms)	10

Temperatures are continuously monitored via high speed pyrometers on each side of the sample. Once the sample has equilibrated, at the target temperature, the front surface of the sample is exposed to a power ramp or laser pulse of around 10 ms. Upon initiation of the laser pulse the pyrometers are triggered and the resulting temperature increase with respect to time is recorded on both the front and rear sides of the sample. The voltage readings are acquired via a General Purpose Interface Bus (GPIB) box, which transfers the data to a personal computer. These are then converted to radiance temperature readings. Using Wien’s law and the measured spectral emissivity, radiance temperature is converted to black-body temperature. Finally, a FFT (Fast Fourier Transform) filter is applied to the experimental data, in order to cut-off high frequency noise via a top hat low band pass filter^17^.

A finite element analysis (FEA) model has been developed to describe the heat transfer conditions during the experiment and is described in previous work^17,18^. The model thermograms are fitted to experimental transients, with thermal conductivity, specific heat and total hemispherical emissivity as fitting parameters. The minimum least square difference between model data and experimental data is found using the Levenberg-Marquardt algorithm. It is important to note that the model has been designed to take into account vaporisation as a heat loss mechanism, based on the equilibrium partial pressures of the various gaseous species emerging on the surface of the ThO_2_ samples. While the methodology for calculating this heat loss mechanism is described elsewhere^18^, the partial pressure calculations can be found in Appendix A (see Supplementary information).

The material properties of ThO_2_ used as input for the FEA model are summarized in Table 2. The emissivity at 645 nm is used for converting measured radiance temperature to true temperature, while the emissivity at 1064 nm governs the portion of laser radiation absorbed by the sample. The emissivities at these two wavelengths have been assumed identical. This assumption is confirmed by the results of Ronchi *et al*.^11^ These authors report temperature dependent emissivity values for ThO_2_ at 960 nm (i.e. close to 1064 nm) and their results are in very good agreement with the current reported values at 645 nm. All thermal conductivity values are corrected to 95% theoretical density using the Barndt and Neuer correction^20,21^. The sample stoichiometry was characterized, via X-ray diffraction, for two samples before and after the laser flash experiments; the O/Th ratio was consistently determined to be 2.000 ± 0.001.

2 $$f(T)=\{\begin{array}{ccc}0.1 & for & T < 1545\,K\\ 3.674\times {10}^{-11}{T}^{3}-6.089\times {10}^{-7}{T}^{2}+2.718\times {10}^{-3}T-2.782 & for & 1545\,K < T < 3000\,K\\ 0.88 & for & T > 3000\,K\end{array}$$

**Table 2:** Material properties of ThO_2_ used as input in the FEA model.

Property	Expression	Reference
linear thermal expansion E (T)*	9.9729 × 10^−10^T ^2^ + 7.8410 × 10^−6^T	Hoch^22^
density ρ(T)	ρ_298_{1+E}^−3^	Hoch^22^
ℇ_(1064 nm)_**	*f*(*T*)	this work
ℇ_(645 nm)_**	*f*(*T*)	ths work

*Fitted to reference experimental data.

**See equation 2.

## Results

### Experimental results of melting point, specific heat, thermal conductivity, emissivity and thermal diffusivity

**Figure 2:**
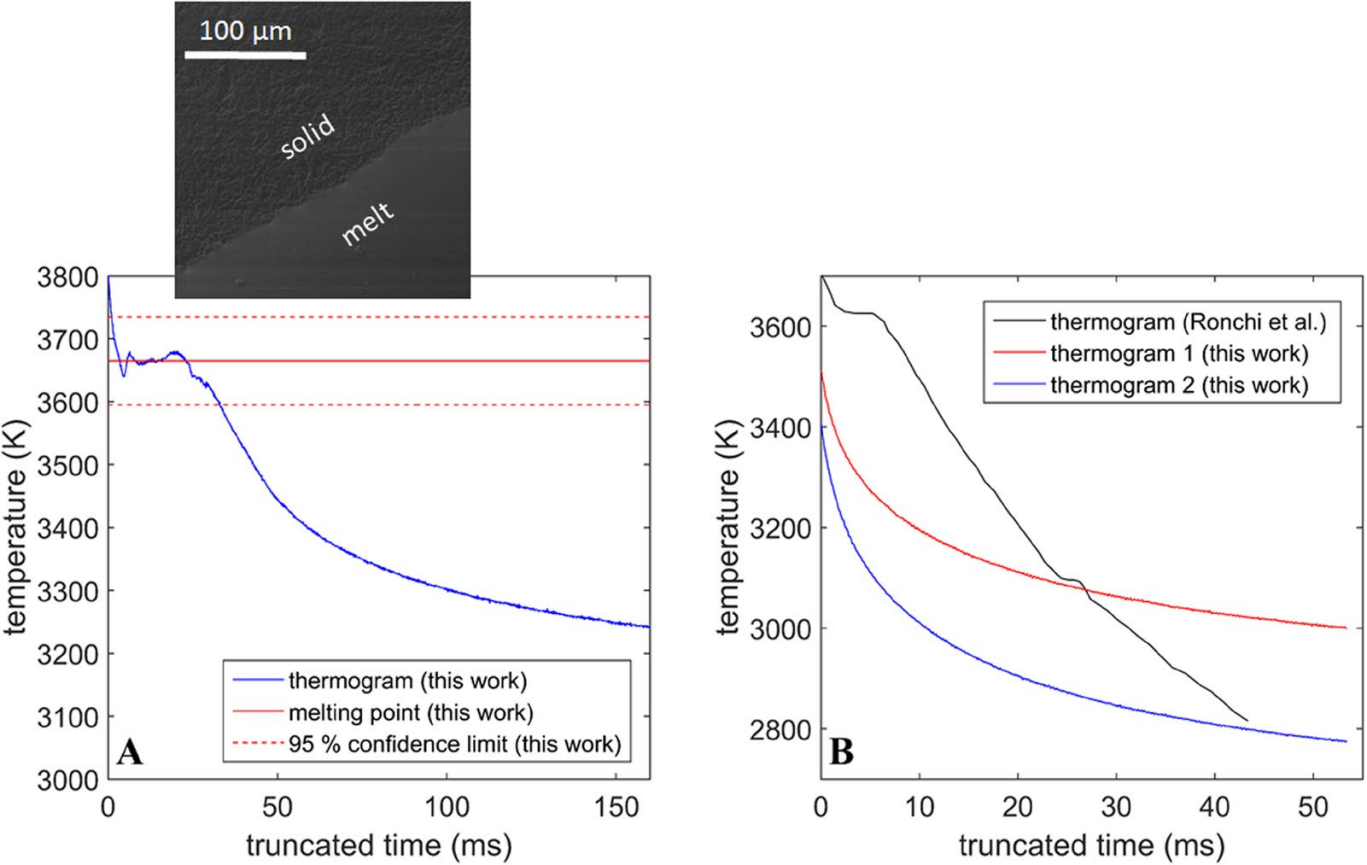
(**A**) Temperature vs. truncated time showing the melting plateau of ThO_2_ via a solid red line and a SEM image of the melting pool presented above the red line. Dashed red lines indicate the confidence limits of the measured melting point. (**B**) Temperature vs. truncated time plots for two thermograms measured in this work compared to the thermogram reported by Ronchi *et al*.^11^.

The melting point of ThO_2_ was identified as 3665 K ± 70 K in this work, which is shown in Fig. 2A. Furthermore, two different cooling curves were recorded and in Fig. 2B compared to the thermogram reported by Ronchi *et al*.^11^. The latter indicates both a melting plateau (at around 3650 K) as well as a pre-melting transition around 3100 K. The cooling curves recorded in this work, however, do not indicate the presence of a pre-melting transition.

**Figure 3:**
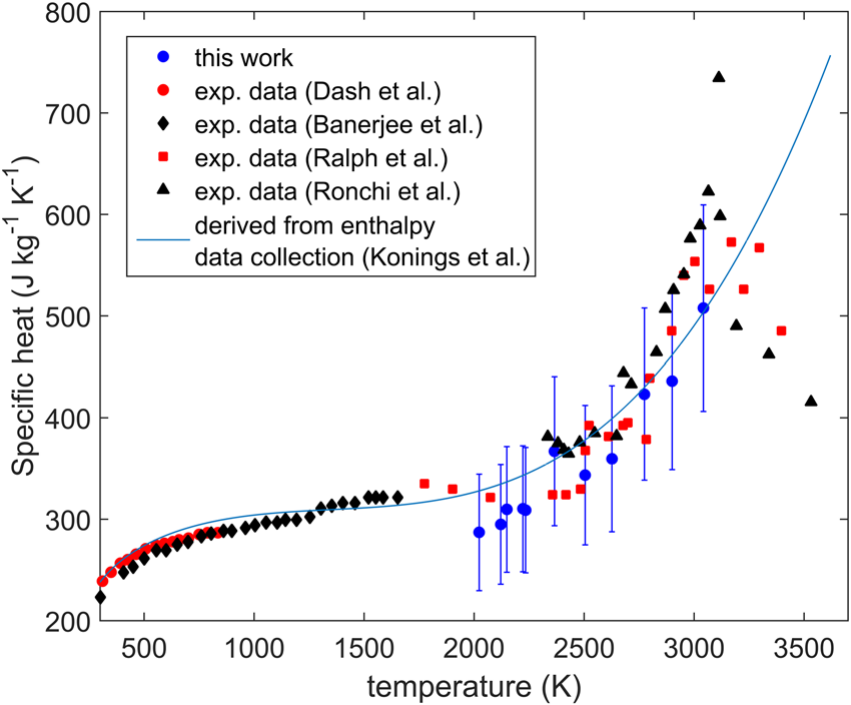
Specific heat evaluated in this work compared to the measurements of Dash *et al*.^9^, Banerjee *et al*.^10^, Ralph *et al*.^12^ and Ronchi *et al*.^11^. The blue line is the recommended curve by Konings *et al*.^23^, based on the derivative of a fit to enthalpy data collected from literature. Error bars correspond to a relative error of two standard deviations.

Figure 3 shows the current measurements of specific heat compared to the experimental data of various authors^11^ and the function describing c_p_(T) recommended by Konings *et al*.^23^. There is generally good agreement between the new results, previous experimental studies and the recommended function up to 3000 K.

**Figure 4:**
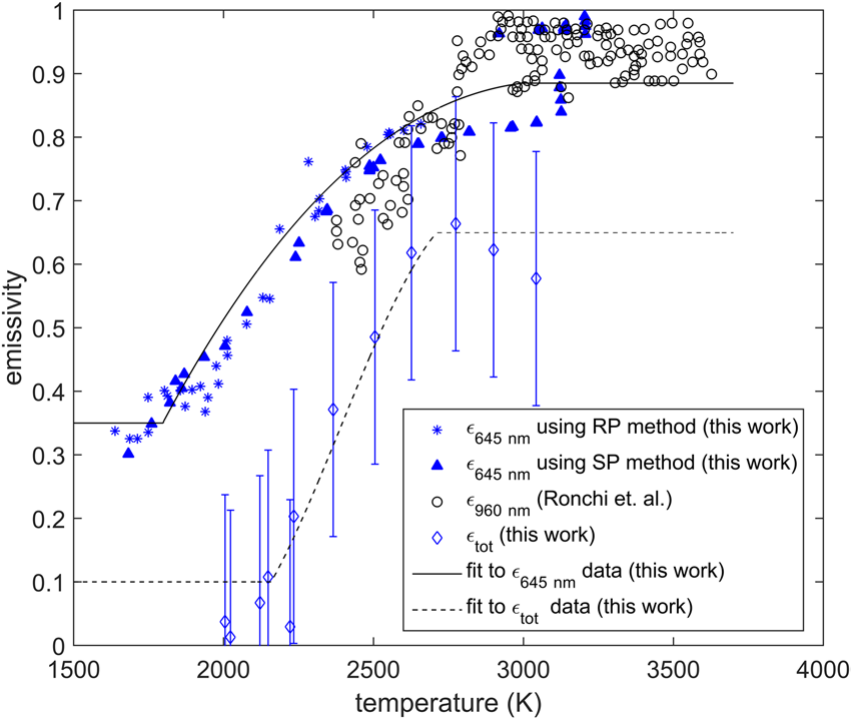
Spectral emissivity at 645 nm and total hemispherical emissivity as a function of temperature measured in this work. Spectral emissivity is compared to the measurements by Ronchi et al .^11^ at 960 nm. The solid and dashed lines are fitted to the data in this work for guidance. Error bars correspond to a relative error of two standard deviations.

Figure 4 presents the spectral and total hemispherical emissivity of ThO_2_. Furthermore, the spectral emissivity values were determined via the two different techniques (the RP and SP methods), which are shown to be consistent with each other. Values for the spectral emissivity measured by Ronchi *et al*.^11^, in the near infrared wavelength range, are close to the measurements presented in the current study, which are in the visible wavelength spectrum. This suggests either a small or no variation of the spectral emissivity of ThO_2_ with respect to wavelength in the range 600–1000 nm. Furthermore, both spectral and total hemispherical emissivity values increase as a function of temperature. The spectral emissivity at 645 and 960 nm is systematically higher compared to the total hemispherical emissivity at all measured temperatures.

**Figure 5:**
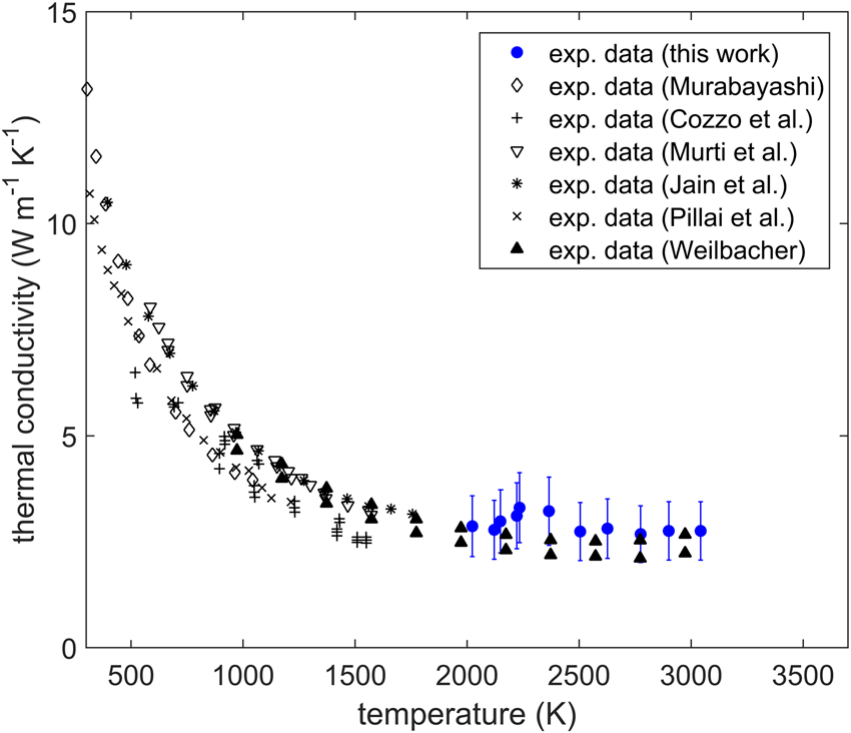
Thermal conductivity as a function of temperature for ThO_2_ corrected to 95% TD. Measurements in this work compared to the data of Murabayashi^14^, Cozzo *et al*.^13^, Murti *et al*.^25^, Jain et al^26^, Pillai *et al*.^24^ and Weilbacher^15^. Error bars correspond to a relative error of two standard deviations.

Figure 5 shows the thermal conductivity measurements for 95% dense ThO_2_ compared to previous values reported by various authors^13–15,24–26^. Most of the literature thermal conductivity values have been indirectly calculated from the thermal diffusivity measurements of the respective authors, the density (based on the thermal expansion recommendation of Hoch and Momin^22^) and the specific heat recommendation of Konings *et al*.^23^ Only the current measurements and data reported by Pillai *et al*.^24^ can be referred to as direct measurements (this is further discussed in section 4.3). All values have been amended using the correction due to Brandt and Neuer^20,21^ to 95% dense ThO_2_. There is good agreement between the existing experimental literature values and the new results. Thermal conductivity decreases from around 9 W m^−1^ K^−1^ at 500 K to approximately 3 W m^−1^ K^−1^ at 2000 K. Beyond 2000 K, the property appears to remain invariant.

**Figure 6:**
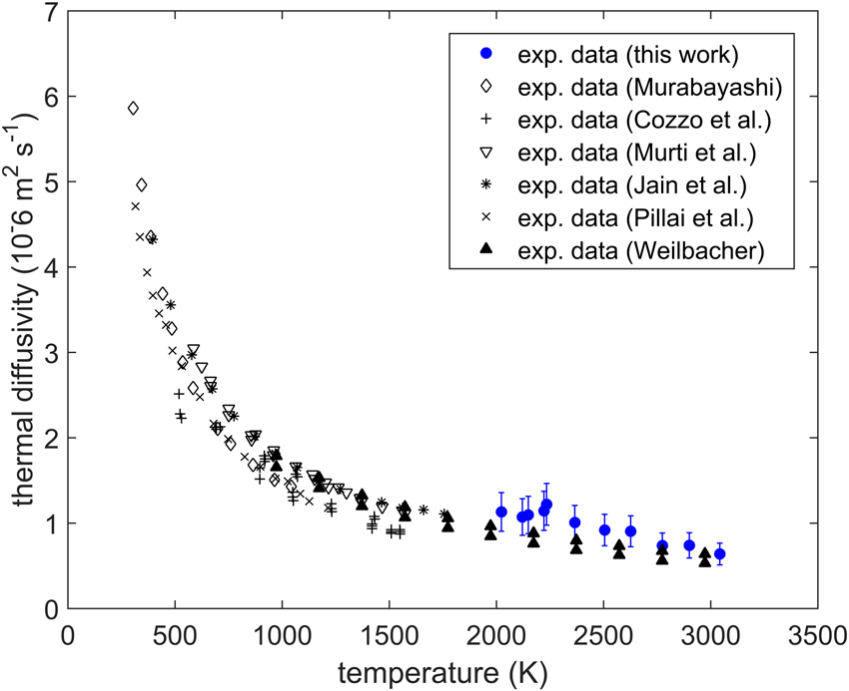
Thermal diffusivity as a function of temperature measured in this work compared to the experimental measurements of Murabayashi^14^, Cozzo *et al*.^13^, Murti *et al*.^25^, Jain *et al*.^26^, Pillai et al^24^. and Weilbacher^15^. Error bars correspond to a relative error of two standard deviations.

Thermal diffusivity as a function of temperature is shown in Fig. 6. The current results are in good agreement with previous values at around 2000 K. In the temperature range 2000–2600 K the new measurements are around 10–15% higher compared to Weilbacher’s results^15^.

### Defect concentrations

Defect concentrations are calculated based on the following reactions:

3 $$Oxygen\,Frenkel\,pairs:{O}_{0}^{x}\to {V}_{O}^{\cdot \cdot }+{O}_{i}^{^{\prime\prime} }$$

4 $$Schottky\,trios:2{O}_{O}^{x}+{Th}_{Th}^{x}\to 2{V}_{O}^{\cdot \cdot }+{V}_{Th}^{{\rm{^{\prime} }}{\rm{^{\prime} }}{\rm{^{\prime} }}{\rm{^{\prime} }}}$$

**Figure 7:**
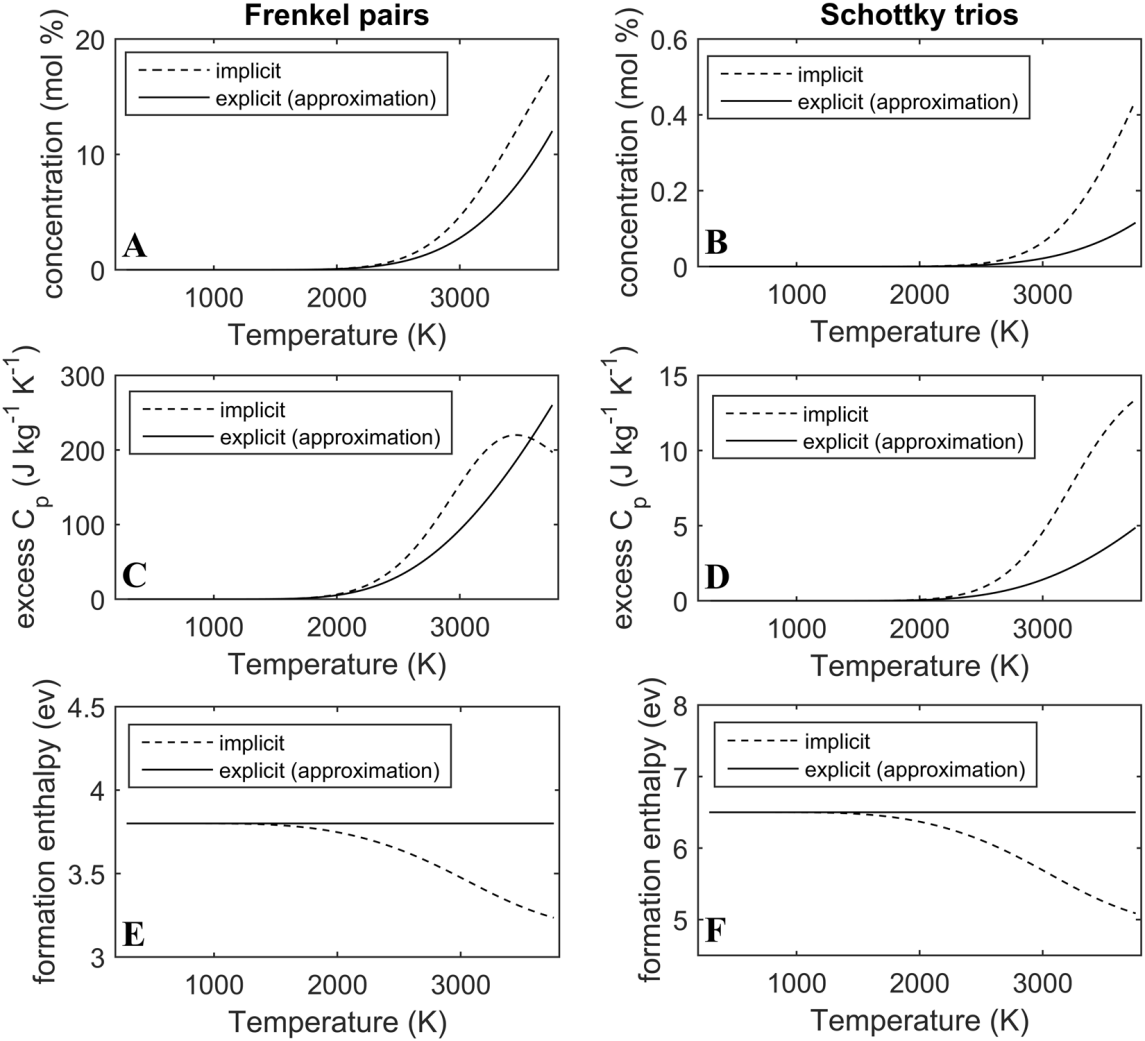
Plot of defect type (the title of each column) vs. the respective concentrations, excess specific heat and formation enthalpies. (**A**,**C**,**E**) represent the temperature dependent concentration, excess specific heat and formation enthalpy of oxygen Frenkel pairs respectively, while (**B**,**D**,**F**) show the temperature dependent concentration, excess specific heat and formation enthalpy of Schottky trios.

From statistical thermodynamics the following non-linear equations are obtained:

5 $${{n}_{F}}^{2}=(\frac{2}{3}-{n}_{F}-2{n}_{S})(\frac{1}{3}-{n}_{F})\,exp(-\frac{{\rm{\Delta }}{H}_{F,Fr}}{{C}_{B}T}+\frac{{\rm{\Delta }}{S}_{NC,Fr}}{{C}_{B}})$$

6 $${{n}_{S}}^{3}={(\frac{2}{3}-{n}_{F}-2{n}_{S})}^{2}(\frac{1}{3}-{n}_{S})exp(-\frac{{\rm{\Delta }}{H}_{F,Sch}}{{C}_{B}T}+\frac{{\rm{\Delta }}{S}_{NC,Sch}}{{C}_{B}})$$

where *n*_*F*_ and *n*_*sch*_ are the Frenkel pair and Schottky trio concentrations per mole of ThO_2_, ${\rm{\Delta }}{H}_{F,Fr}$ and ${\rm{\Delta }}{H}_{F,Sch}$ are the formation enthalpies of Frenkel and Schottky defects respectively, while ${\rm{\Delta }}{S}_{NC,Fr}$ and ${\rm{\Delta }}{S}_{NC,Sch}$ are the non-configurational entropies associated with the same Frenkel and Schottky defect species. The details of the calculation methods are described in a previous work^18^, while the defect model input parameters can be found in Table 3. However, it should be noted that the formation enthalpy is concentration dependent, due to the net attraction between oppositely charged defect species, leading to the implicit solution. The (approximate) explicit form of this equation is obtained by neglecting defect concentrations in the pre-exponential terms of equations (5) and (6) and also by assuming no columbic defect interactions. The results from equations (5) and (6) have been plotted in Fig. 7. At temperatures below 2300 K the two methods yield similar results, however, at higher temperatures the implicit solution calculates higher defect concentrations compared to the explicit approximation. Furthermore, the implicit results show a maximum in the excess specific heat due to Frenkel pairs and a reduction in enthalpy (see Fig. 7C). Schottky trios exhibit more significant relative differences, between the two solutions, compared to Frenkel pairs (see Fig. 7).

**Table 3:** Summary of input parameters for the defect concentration calculation.

Parameter description	Units	Symbol	Value	Indicative Range	Ref.
Frenkel pair formation enthalpy	eV	${\rm{\Delta }}{{\boldsymbol{H}}}_{{\boldsymbol{F}},{\boldsymbol{Fr}}}$	3.8	3.0–4.5	^27–30^
Schottky trio formation enthalpy	eV	${\rm{\Delta }}{{\boldsymbol{H}}}_{{\boldsymbol{F}},{\boldsymbol{Sch}}}$	6.5	6.0–8.0	^27,28^
Frenkel pair formation entropy	meV K^−1^	${\rm{\Delta }}{{\boldsymbol{S}}}_{{\boldsymbol{NC}},{\boldsymbol{Fr}}}$	0.78	—	^29^
Schottky trio formation entropy	meV K^−1^	${\rm{\Delta }}{{\boldsymbol{S}}}_{{\boldsymbol{NC}},{\boldsymbol{Sch}}}$	0.17	—	**

**Recommended in this work.

### Specific heat and melting point

**Figure 8:**
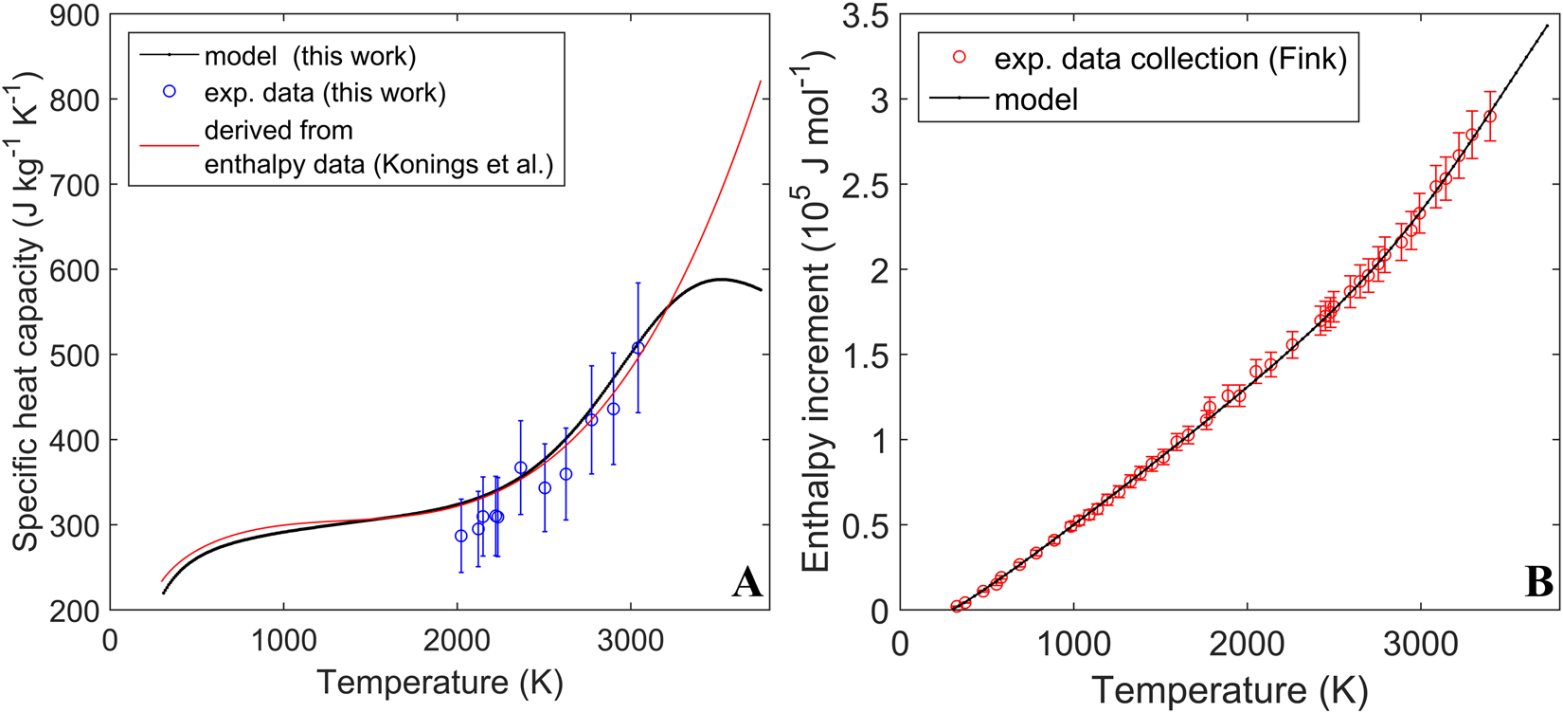
(**A**) Measurements of specific heat as a function of temperature compared to the model results and the fit recommended by Konings *et al*.^23^ (**B**) Enthalpy increment data collected by Fink^31^ compared to the model output. Error bars correspond to a relative error of two standard deviations.

The specific heat of ThO_2_ can be understood in terms of the following terms:

7 $${c}_{P}^{tot}={c}_{V}^{h}+{c}_{P}^{d}+{c}_{V\to P}$$

where ${c}_{P}^{tot}\,$ is the total specific heat at constant pressure, ${c}_{V}^{h}\,$is the harmonic contribution to specific heat at constant volume, ${c}_{P}^{d}$ is defect term of specific heat at constant pressure and ${c}_{V\to P}$ is the an-harmonic term of specific heat needed to convert specific heat at constant volume to specific heat at constant pressure. The methodology for modeling specific heat has been described previously^18^. Figure 8A compares the experimental data measured in this work with the model results and the function recommended by Konings *et al*.^23^. It shows that both the model and the recommended function agree with the current measurements. The differences between the model and the recommended relationship for specific heat are significant prior to melting, where the model predicts lower specific heat values compared to the results obtained by extrapolating the recommended empirical function. Even though not plotted in Fig. 8A, the model also predicts the specific heat of ThO_2_ at lower temperatures to be increasing from 0 at 0 K to ≈210 J kg^−1^ K^−1^ at 300 K. Figure 8B compares the model results for enthalpy increment to the experimental data set collected by Fink^31^. The model results are in excellent agreement with experiment.

### Thermal expansion

**Figure 9:**
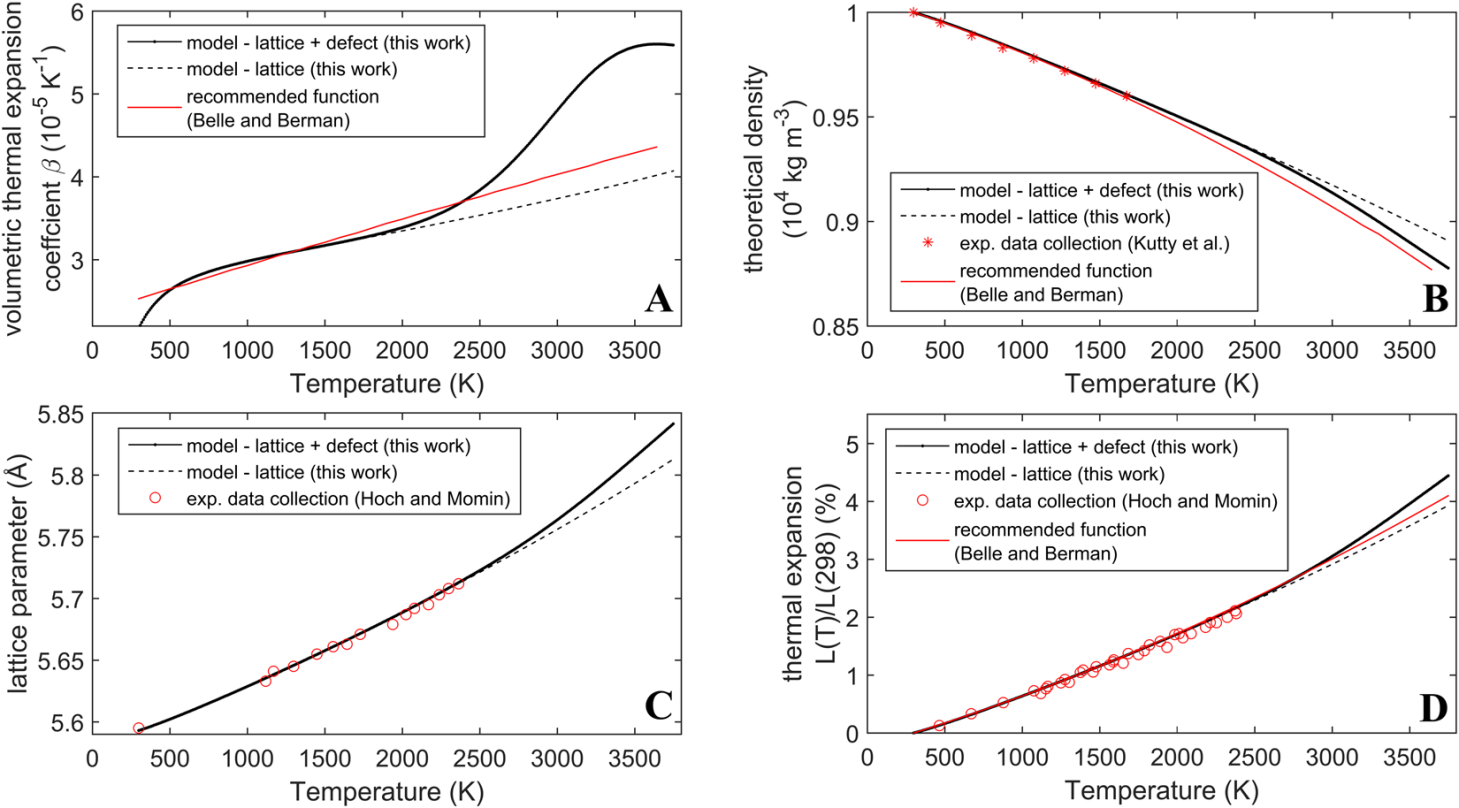
Experimental and model results for various dimensional property parameters of ThO_2_ as a function of temperature. (**A**) Shows the volumetric thermal expansion coefficient as a function of temperature; (**B**) the theoretical density as a function of temperature; (**C**) lattice parameter as a function of temperature and (**D**) thermal expansion vs. temperature. The model results have been compared to available experimental data^4^ and the recommended correlation^22^. The dashed black lines show only the lattice contribution to thermal expansion, while the solid black lines represent the combined contributions of lattice and defects.

The coefficient of volumetric thermal expansion coefficient can be understood in terms of the following contributions:

8 $${\beta }^{tot}={\beta }^{l}+{\beta }^{d}$$

where ${\beta }^{tot}$, ${\beta }^{l}$, ${\beta }^{d}$ are the total, lattice and defect contributions respectively. These terms are modeled using the methodology described previously^18^. The defect term is based on the formation enthalpy of the defect species, while the lattice term utilizes the specific heat at constant volume computed from the phonon density of states model (see Appendix B provided as part of the supplementary information). Both defect and lattice terms use experimental values for the temperature dependent bulk modulus. Figure 9B,C and D show that the model results agree well with available experimental data up to 2300 K. However, more experimental data is needed at higher temperatures to validate the defect model prediction. Figure 9A shows that above 2300 K the coefficient of volumetric thermal expansion calculated by the model increases significantly faster with respect to temperature compared to the recommended function by Belle and Berman^4^. Figure 9B shows that the calculated density in this work is higher compared to the proposed correlation by Belle and Berman^4^ above 1500 K. In Fig. 9D the model predictions are shown to exceed the correlation proposed by Belle and Berman at temperatures above 2500 K. Finally, all subplots in Fig. 9 show that between 2000 K and the melting point of ThO_2_ (approximately 3665 K), the defect contribution becomes increasingly more significant, which is similar to previously reported results for UO_2_^18^.

### Thermal conductivity

In this section an expression will be proposed for the temperature dependence of thermal conductivity of ThO_2_. The total thermal conductivity k_tot_, is:

9 $${k}_{tot}={k}_{l}+{k}_{el}$$

where *k*_*l*_ is the lattice contribution and *k*_*el*_ is the electronic contribution. The lattice contribution has been determined empirically and mechanistically. The mechanistic approach or model has previously been applied to UO_2_ and is described in detail elsewhere^18^. It must be noted, that in the case of ThO_2_, spin-phonon scattering is not considered. This is consistent with the absence of magnetic ordering in ThO_2_ (the material is diamagnetic). The parameters used for the calculation of thermal conductivity are summarised in Table 4.

**Table 4:** Summary of input parameters for the calculation of lattice thermal conductivity of ThO_2_.

Parameter description	Units	Symbol	Value	Indicative Range	Ref.
Grüneisen parameter	**—**	*γ*	1.9	**—**	**
atomic mass	u	${{\boldsymbol{M}}}_{{\boldsymbol{at}}}$	264	**—**	**—**
grain size	µm	${{\boldsymbol{L}}}_{{\boldsymbol{g}}}$	10.0	8–12	*
pore fraction	(%)	${{\boldsymbol{v}}}_{{\boldsymbol{p}}}$	7	**—**	*
molar mass	kg mol^−1^	${{\boldsymbol{M}}}_{{\boldsymbol{mol}}}$	0.264	**—**	**—**
lattice parameter (298 K)	Å	${{\boldsymbol{a}}}_{0}$	5.597	**—**	
density (theoretical)	kg m^−3^	${\boldsymbol{\rho }}$	10000	**—**	**—**
Bulk modulus (298 K)	GPa	**K**_**0**_	200	175–230	^36,37^

*Determined experimentally in this work.

**Recommended in this work.

**Figure 10:**
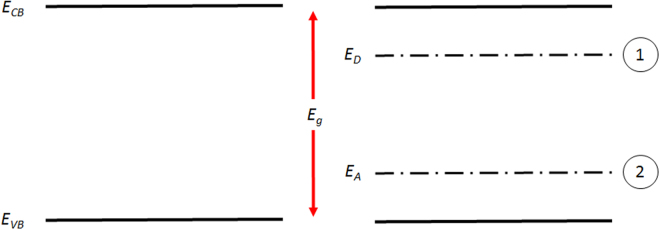
Schematic of the ThO_2_ band gap free of defects (left) and the possible donor (1) or acceptor energy levels (2) (right). E_CB_ is the energy of the conduction band edge, E_VB_ is the valence band edge energy, while E_A_ and E_D_ are the possible acceptor and donor energy levels, respectively.

The electronic term in equation 9 has been determined empirically based on the following assumptions: (i) significant defect concentrations are generated at high temperatures, which lead to the formation of isolated energy states inside the band gap of ThO_2_ (see Fig. 10); (ii) these defect states lead to a shift in the Fermi energy towards either the conduction or valence bands; (iii) hybridization exists between Th-f and O-p orbitals (i.e. excited charge carriers are free and not localized as they are for UO_2_).

According to Yang^32^, the thermal conductivity due to the excitation of an electron or hole in a semi-conductor can be expressed by:

10 $${k}_{el}=\sigma T{(\frac{{C}_{B}}{e})}^{2}(2+{[4+\frac{{E}_{DF}}{{C}_{B}T}]}^{2}\frac{{\mu }_{e}}{{\mu }_{h}}{(1+\frac{{\mu }_{e}}{{\mu }_{h}})}^{-2})$$

where *σ* is electrical conductivity (Ω^−1^ m^−1^), *T* is temperature (K), *C*_*B*_ is Boltzmann’s constant (eV K^−1^), *e* is the elementary charge (C), *E*_*DF*_ is the electron formation energy (eV), due to defect donor or acceptor levels, *μ*_*e*_ is the electron mobility, *μ*_*h*_ is the hole mobility. Furthermore, in the case of electron carriers, ${E}_{DF}={E}_{CB}-{E}_{F}$, while for holes ${E}_{DF}={E}_{F}-{E}_{VB}$, where *E*_*F*_, *E*_*CB*_, *E*_*VB*_ are the Fermi energy, conduction band energy and valence band energy, respectively. Here, it will be assumed that electron and hole mobilities are equal, so that:

11 $${k}_{el}=\sigma T{(\frac{{C}_{B}}{e})}^{2}(2+4{[1+\frac{{E}_{DF}}{4{C}_{B}T}]}^{2})$$

12 $${k}_{el}=\sigma T{(\frac{{C}_{B}}{e})}^{2}(6+2[\frac{{E}_{DF}}{{C}_{B}T}]+\frac{1}{4}{[\frac{{E}_{DF}}{{C}_{B}T}]}^{2})$$

13 $$\sigma =nq\mu $$

14 $$n\propto \exp (-\frac{{E}_{DF}}{{C}_{B}T})$$

**Figure 11:**
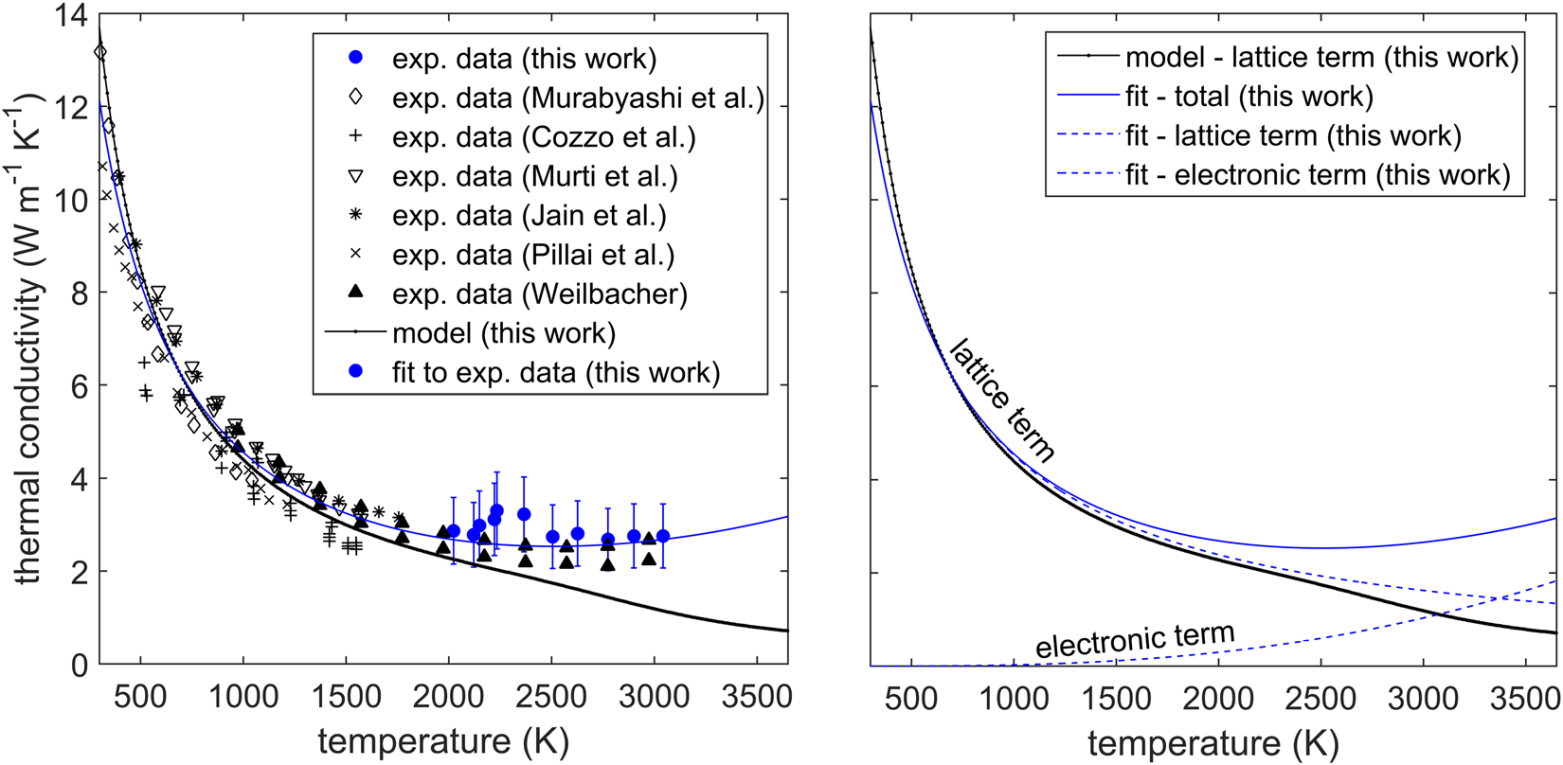
(**A**) Thermal conductivity as a function of temperature measured in this work compared to: the experimental data of Murabayashi^14^, Cozzo *et al*.^13^, Murti *et al*.^25^, Jain *et al*.^26^, Pillai *et al*.^24^, Weilbacher^15^, the current model results and the fit proposed in this work. (**B**) The lattice term of thermal conductivity calculated by the model compared to the lattice and electronic terms obtained from the fit (the word model refers to the mechanistic approach used to calculate lattice thermal conductivity, which has been described previously^18^). Error bars correspond to a relative error of two standard deviations.

Assuming that free carrier scattering is dominated by impurities^33,34^, since at high temperatures the concentration of charged defects (oxygen Frenkel pairs) is significant, the mobility can be approximated via:

15 $$\mu \propto {T}^{3/2}$$

16 $${\sigma }_{el}=C{T}^{3/2}\exp (-\frac{{E}_{DF}}{{C}_{B}T})$$

where *C* is a constant of proportionality. Therefore, thermal conductivity will be expressed by the following expression:

17 $${k}_{tot}=\frac{1}{A+BT}+C{T}^{5/2}{(\frac{{C}_{B}}{e})}^{2}(6+2[\frac{{E}_{DF}}{{C}_{B}T}]+\frac{1}{4}{[\frac{{E}_{DF}}{{C}_{B}T}]}^{2})\exp (-\frac{{E}_{DF}}{{C}_{B}T})$$

where A and B are constants associated with the empirical lattice term of thermal conductivity. The consatnts *C* and *E*_*DF*_ are associated with the electronic term of thermal conductivity. This expression has been fitted to the experimental data measured in this work as well as to literature data. The fitting results suggest that: $A=2.31692\times {10}^{-2}{{\rm{W}}}^{-1}mK;$ $B=1.97516\times {10}^{-4}{{\rm{W}}}^{-1}m;$ $C=7.4086228\times {10}^{-2}\,{{\rm{C}}}^{2}\,{{\rm{J}}}^{-1}{s}^{-1}{m}^{-1}{{\rm{K}}}^{-3/2};$ ${E}_{DF}=0.173227\,eV.$ This value is far lower compared to the activation energies recommended by Bates^35^. Figure 11 summarises and compares the resulting fit, the new experimental results presented in this work, existing literature data and the model prediction of the thermal conductivty of ThO_2_ (the mecahnistic model has been established in  a previous study^18^, while model input parameters are provided in Table 4).

### Emissivity

Based on the work of Mason^38^ and Flügge^39^, the spectral emissivity of ThO_2_ transitions from a transparent state (0–0.2) to an opaque state (0.85–0.95) above a wavelength of approximately 7 *μm* (measurements by Mason were performed at 1273 K). At longer wavelengths (above 7 *μm*) or alternatively lower photon energies, absorption and emission can be understood in terms of the interaction of photons with phonons. In this work it will be assumed that below a critical wavelength of 2 $\mu m$ the emissivity is temperature dependent. This is consistent with equation (2) and can be explained by the formation of intrinsic donor states (represented by E_D_ in Fig. 10) or acceptor states (represented by E_A_ in Fig. 10), in the band gap of ThO_2_. Based on the work of Lu *et al*.^27^, it is likely that these defect energy levels are indeed donor states formed via the creation of oxygen interstitials, as their respective energy level is in relative proximity to the conduction band edge of ThO_2_. Furthermore, oxygen Frenkel pairs are also the dominant defect species at elevated temperatures. In an intermediate wavelength range 2–7 $\mu m$, it will be assumed that the material remains transparent with a spectral emissivity of 0.1, where the photon energy is too high for any interaction with lattice vibrations to be possible and too low for an electron to be excited from the donor states to the conduction band. Finally, if photons have an energy higher than 6 eV (approx. 0.2 µm) it is considered that electrons can be excited directly from the valence to the conduction band in ThO_2_. These considerations yield the following dependence of emissivity on wavelength and temperature:

18 $${\epsilon }(\lambda ,T)\{\begin{array}{lll}0.95 & for & \lambda  > 7\mu m\\ 0.05 & for & 2\mu m < \lambda  < 7\mu m\\ f(T) & for & 0.2\mu m < \lambda  < 2\mu m\\ 0.95 & for & \lambda  < 0.2\mu m\end{array}$$

An expression for total hemispherical emissivity can be derived from Planck’s law leading to the following expression:

19 $${{\epsilon }}_{tot}(T)=\frac{{\int }_{0}^{\infty }{\epsilon }(\lambda ,T){\lambda }^{-5}{(\exp (\frac{{c}_{2}}{\lambda T})-1)}^{-1}d\lambda }{{\int }_{0}^{\infty }{\lambda }^{-5}{(\exp (\frac{{c}_{2}}{\lambda T})-1)}^{-1}d\lambda }$$

**Figure 12:**
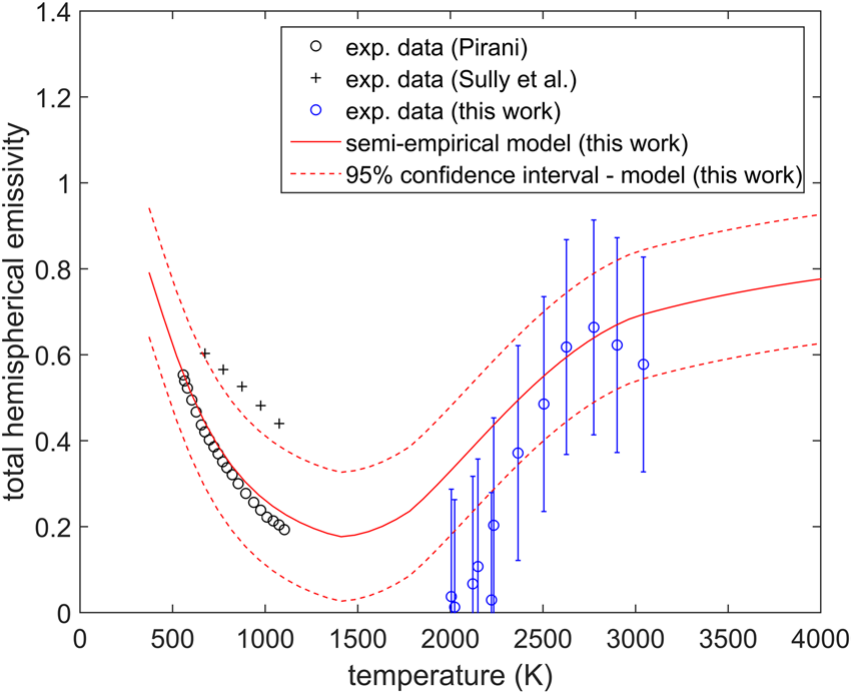
Total hemispherical emissivity measured in this work plotted together with the experimental results of Pirani^41^, the measurements by Sully *et al*.^40^ and the semi- empirical model proposed in this work. The red dashed lines represent the indicative 95% confidence intervals of the model calculation. Error bars correspond to a relative error of two standard deviations.

Figure 12 shows the measured total hemispherical emissivity in this work plotted together with the few existing data sets for this property at lower temperatures (500–1300 K), all of which are compared to the calculation obtained by numerically solving equation (18). There is good agreement between the model and the experimental data of Sully *et al*.^40^ as well as the measurements in this work above 2400 K. There is also qualitative agreement between the trends observed experimentally and the ones appearing from the model prediction. Total hemispherical emissivity decreases between 500 K and 1400 K. Just below 1500 K a minimum is reached, followed by an accelerated increase with respect to temperature up to ~2800 K. Above 2800 K total hemispherical emissivity increases at a slower rate with respect to temperature.

## Discussion

### Defects

Figure 7 shows significant differences at high temperatures between the two approaches for calculating defect concentrations – the implicit solution and explicit approximation. The implicit method calculates higher defect concentrations compared to the explicit one, as the prior includes the net attractive coloumbic potential between oppositely charged species. Frenkel pairs dominate over Schottky trios due to their significantly lower formation enthalpy and high non-configurational entropy. The maximum in the excess specific heat is thus due to oxygen Frenkel pair formation and can be understood in terms of an onset of saturation of oxygen interstial sites (also discussed previously^18^).

### Specific heat, melting and pre-melting transition

The new measurements of specific heat are generally in good agreement with the works of Ralph^12^ and Ronchi *et al*.^11^, they do not, however, indicate the abrupt increase in this propety at around 3100 K as measured by Ronchi *et al*.^12^. This is further supported by the cooling curves (see Fig. 2B), which clearly show the absence of a pre-melting transition. This could be due to the nature of the set-up used by Ronchi *et al*.^11^. In their work, a spherical ThO_2_ sample of 1 mm diameter was held in place by a Tungsten pin and was melted via pulsed laser heating. The maximum temperature recorded during heating was reported to be 4500 K. This means that the temperature reached on the surface of the sample was approximately 800 K above the melting point of Tungsten (around 3700 K). It is thus possible that not only the ThO_2_ sample was melted but also the Tungsten pin. This means that the two molten materials could have interdiffused leading to the formation of a ThO_2_-W mixture. Upon cooling this mixture could have undergone a eutectic transition, which was at a lower temperature. This would explain the pre-melting plataeu measured by Ronchi *et al*.^11^ at ~3100 K.

As discussed in section 3.3 the model predicts that the specific heat of ThO_2_ increases rapidly up to 500 K (see also Fig. 8A). This is due to lattice vibrations, in particular, due to phonons accessing new higher energy (or frequency) modes of vibration, as well as due to the increase in population of existing high frequency phonon states. In the temperature interval of 1000 K to 2000 K the steady increase in specific heat is predominantly governed by the thermal expansion of the material. Above around 2300 K specific heat begins to increase again at a higher rate with respect to temperature. This phenomenon can be explained by defect production, in particular the creation of oxygen Frenkel pairs with a limited contribution from Schottky trios. The faint maximum predicted by the model prior to melting is a result of the contraint the lattice imposes on the formation of oxygen interstials^18^.

### Thermal conductivity

The work of Pillai *et al*.^24^ is the only previous study providing direct measurements of thermal conductivity. The new measurements of the thermal conductivity of ThO_2_ above 2000 K are the first direct laser flash measurements of this property at such high temperatures. The current measurements are characterised as direct, since specific heat values are not taken from literature, but simultaneously measured. It must be noted that in the current work density is still a necessary input for the FEA model. However, since density and specific heat are completely correlated (correlation coefficient of unity), any small or moderate uncertainty in density would not impact the results of thermal conductivity as significantly as the values of specific heat. Thus, the experimental technique used here can still be considered a predominantly direct method of measuring thermal conductivity. In comparison, the measurements of Weilbacher^15^ can be refered to as indirect, as they utilize the measured thermal diffusivity, together with literature values of specific heat^23^ and density^22^, in order to obtain thermal conductivity. Nevertheless, the new results are in good agreement with Weilbacher’s results. There is, however, no evidence in the new measurements for a sudden increase in thermal conductivtiy around 3000 K as was discussed by Kutty *et al*.^7^.

Figure 11A shows a decrease in thermal conductivity in the temperature range 300 K to 2000 K. This occurs as a result of various phonon scattering mechanisms - grain boundary scattering, phonon- phonon scattering and defect scattering. Among these, the phonon- phonon interaction dominates within this temperature range. At temperatures above 2000 K the model predicts an accelerated thermal conductivity degradation (see Fig. 11A), due to the significant increase in the concentration of defects. This decrease is, however, compensated by the mobilisation of free charge carriers from intra-band, intrinsic donor or acceptor states. These are assumed to be associated with charged defects and in particular oxygen Frenkel pairs, due to their significant concentration at elevated temperatures. The competition between the decreasing lattice thermal conductivity and the growing electronic term leads to the weak minimum at 2900 K. A function is suggested in this study for the variation of thermal conductivity with respect to temperature. This function provides a good fit to all new and existing data, however, it should be noted that further work is necessary in order to quantify and understand the  activated thermo-electronic transport mechanism. In particular the discrepancy between the electronic activation energy reported here and the activation energies reported by Bates^35^ must be examined in the future.

### Total hemispherical emissivity

The new results of total hemispherical emissivity, even though approximate, are the first results of this property at high temperatures for ThO_2_. It has been shown by both Sully *et al*.^40^ and Pirani^41^ that total hemispherical emissivity decreases between 300 K and 1300 K. This can be understood in terms of a shift of the Planck curve to shorter wavelengths (or higher energies) with increasing temperature. At temperatures up to 1500 K ThO_2_ is transparent to higher energy photons (i.e. photons in the visible and near infrared wavelength range). Thus, increasing the temperature between 300 K and 1500 K leads to a greater contribution to total hemispherical emissivity from the shorter wavelengths (0.2 *µm* < *λ* < 7 *µm*), at which point the material is nearly transparent to electro-magnetic radiation. As the temperature increases the concentration of defects, in particular oxygen Frenkel pairs, rapidly increases. These defects lead to the formation of “intra-band gap” states, which provide a path for electro-magnetic radiation absorption and emission. In particular, electrons could be promoted either from donor levels to the conduction band or excited from the valence band edge to acceptor states. This mechanism can proceed at energies significantly lower compared to the band gap of ThO_2_, leading to absorption and emission within the visible and very near-infrared wavelength range (0.2 *µm* < *λ* < 2 *µm*). The creation of more defect species leads to an increase in the density of states in these “intra-band gap” states, which then increases the probability of defect assisted photon absorption/emission. This explains the increase in spectral emissivity in the visible wavelength range above 1500 K, which was measured in this work. Moreover, the proposed mechanism is also consistent with the increase in spectral emissivity at 960 nm previously reported by Ronchi *et al*.^11^. Thus, at temperatures above 1500 K there are two competing phenomena, which yield the minimum predicted by the semi-empirical model (shown in Fig. 12): i) the shift of the Planck curve towards a shorter transparent wavelength region with increasing temperature; ii) defect assisted absorption at these shorter wavelengths, which increases with temperature and leads to a shift from transparency to opacity.

### Thermal expansion

The model for the volumetric thermal expansion coefficient of ThO_2_ predicts a significantly different variation of volumetric thermal expansion compared to the empirical correlation by Belle and Bermann^4^. In this work it has been demonstrated theoretically that this property is predominantly governed by lattice vibrations up to 2300 K. Beyond this temperature the formation of defects contributes to the dimensional changes in ThO_2_. Displacement of atoms from their perfect lattice sites leads to an additional increase in the rate of volume change with respect to temperature. This results in an increase in thermal expansion and a decrease in the density of ThO_2_.

### Relevance of new results for nuclear fuel performance

The new experimental and model results have several implications for the performance of ThO_2_ containing nuclear fuels under accident conditions. The thermal conductivity of ThO_2_ has been shown to be relatively high compared to UO_2_ up to 3000 K. This suggests that ThO_2_ bearing fuel will have a higher margin to melting compared to UO_2_. The measurements of total hemispherical emissivity show an increase with temperature, which is also a favourable feature of the material when subject to accident conditions. The increasing total hemispherical emissivity would retard the heating-up of the material. Specific heat has been shown to increase significantly with temperature, which would lead to a beneficial slower heating rate of the fuel. However, it has been predicted (via the defect model), that specific heat would cease to increase prior to melting, reaching a maximum followed by a decrease. This prediction is markedly different from the predictions of extrapolated empirical functions at temperatures above 3000 K. The lower specific heat above 3000 K would lead to a faster heat-up of ThO_2_ bearing fuels. Finally, the coefficient of volumetric thermal expansion above 2500 K, calculated here, is significantly higher compared to the empirically extrapolated values. This implies the fuel would expand more quickly with respect to temperature during extreme off-normal scenarios, resulting in higher tensile stresses exerted by the fuel on the cladding. The compound effect of these differences in property values should be realized through a fuel performance code.

### Summary

Measurements of thermal conductivity, specific heat, and thermal diffusivity, spectral and total hemispherical emissivity of ThO_2_ have been presented in the temperature range 2000 K to 3050 K. Furthermore, solid state physics models have been used in the interpretation of the results. Theory and experiment have led to the following conclusions:

- Measurements show that the specific heat of ThO_2_ increases with temperature up to 3500 K, which can be attributed predominantly to the formation of oxygen Frenkel pairs. The defect model shows that a maximum in specific heat exists around 3500 K, due the onset of saturation of oxygen interstitial sites.
- The experimental results for thermal conductivity show that while this property initially decreases, with increasing temperature, it levels out above 2000 K. Nevertheless, the solid state physics model shows continuous degradation of lattice thermal conductivity above 2000 K. This is, however, compensated by an electronic contribution, most likely due to the thermal delocalization of electrons from donor or acceptor levels. The population of these levels is associated with the rapidly increasing concentration of oxygen defects as a function temperature.
- The model for thermal expansion shows significant defect assisted swelling of ThO_2_ above 2500 K. A maximum in the coefficient of volumetric thermal expansion is predicted prior to melting, due to the onset of saturation of oxygen interstitials.
- Spectral emissivity (in the visible and near infra-red wavelength range) and total hemispherical emissivity were shown to increase above 1500 K. This can be understood in terms of: i) an increase of the population of the electronic density of states of donor/acceptor levels, due to the increasing defect concentration and ii) a simultaneous shift of the Planck curve to lower wavelengths (at which donor/acceptor level assisted absorption takes place).

## Electronic supplementary material

Supplementary information
