# Supplementary material for: High temperature measurements and condensed matter analysis of the thermo-physical properties of ThO2
Source: Sci Rep. 2018 Mar 22;8:5038. doi: 10.1038/s41598-018-21406-w (PMC5864967; doi:10.1038/s41598-018-21406-w)
Supplement: Supplementary file 1 — Supplementary information [file 41598_2018_21406_MOESM1_ESM.docx]

**Supplementary information**

High temperature measurements and condensed matter analysis of the thermo-physical properties of ThO_2_

T. R. Pavlov^1,2^, T. Wangle^3,4^, M. R. Wenman^2^, V. Tyrpekl^3^, L. Vlahovic^1^,

D. Robba^1^, P. Van Uffelen^1^, R. J. M. Konings^1^, and R. W. Grimes^2^

^1^ *European Commission, Joint Research Centre, Institute for Transuranium Elements, P.O. Box 2340, 76125 Karlsruhe, Germany*

*^2^ Department of Materials and Centre for Nuclear Engineering, Imperial College London, Royal School of Mines, London, SW7 2AZ, UK*

^3^*SCK•CEN, Institute of Nuclear Materials Science, Boeretang 200, 2400 Mol, Belgium*

^4^*KU Leuven, Department of Materials Engineering, Kasteelpark Arenberg 44, 3001 Heverlee, Belgium*

A modification of the Rand-Markin model^1,2^ has been used for the calculation of gaseous partial pressures:

${\frac{1}{2}O}_{2}\left( g \right)\to O\left( g \right) p_{O}=exp\left[ \frac{{\Delta G}_{0, 1}}{RT} \right]{p_{O_{2}}}^{1/2}$ (A1)

${ThO}_{2}\left( s \right)\to{ThO}_{2}\left( g \right) p_{{ThO}_{2}(g)}=exp\left[ \frac{{-\Delta G}_{0, 2}}{RT} \right]$ (A2)

${Th\left( g \right)+\frac{1}{2}O}_{2}\left( g \right)\to ThO\left( g \right) p_{Th(g)}=exp\left[ \frac{{-\Delta G}_{0, 3}}{RT} \right]p_{{ThO}_{2}(g)}{p_{O_{2}}}^{-1/2}$ (A3)

$ThO\left( g \right)+{\frac{1}{2}O}_{2}\left( g \right)\to{ThO}_{2}\left( g \right) p_{ThO(g)}=exp\left[ \frac{{-\Delta G}_{0, 4}}{RT} \right]p_{{ThO}_{2}(g)}{p_{O_{2}}}^{-1/2}$ (A4)

$p_{O_{2}}=exp\left[ \frac{{\Delta G}_{0, 5}}{RT} \right]$ (A5)

The standard Gibbs free energies of formation have been refitted, based on the most recent updated information available in the FactSage database^3,4^. These are summarized below in Table A1. Figure A1 compares the relative error between the calculated partial pressures based on equation (A1) to (A5) and the values obtained from FactSage. It should be noted that, due to the use of a very pure oxygen atmosphere during the laser flash experiments, equation (A5) is modified to $p_{O_{2}}=1$when calculating the vaporisation heat losses.

Table A1. Summary of standard formation enthalpies and entropies. The subscript corresponds to the respective reaction number in equations (A1) to (A5).

| ${\Delta H}_{0}$ | Value  (kJ mol^-1^) |  | ${\Delta S}_{0}$ (T) | Function  (kJ mol^-1^) |
| --- | --- | --- | --- | --- |
| ${\Delta H}_{0, 1}$ | 250.5 |  | ${\Delta S}_{0, 1}$ | 1.649 × 10^-3^ × T +61.6 |
| ${\Delta H}_{0, 2}$ | 785.84 |  | ${\Delta S}_{0, 2}$ | 8.423 × 10^-3^ × T – 213.7 |
| ${\Delta H}_{0, 3}$ | -149.9 |  | ${\Delta S}_{0, 3}$ | 4.748 × 10^-4^ × T + 13.21 |
| ${\Delta H}_{0, 4}$ | -406.5 |  | ${\Delta S}_{0, 4}$ | 2.446 × 10^-3^ × T + 46.68 |
| ${\Delta H}_{0, 5}$ | -938.4 |  | ${\Delta S}_{0, 5}$ | -6.743 × 10^-3^ × T + 295.1 |


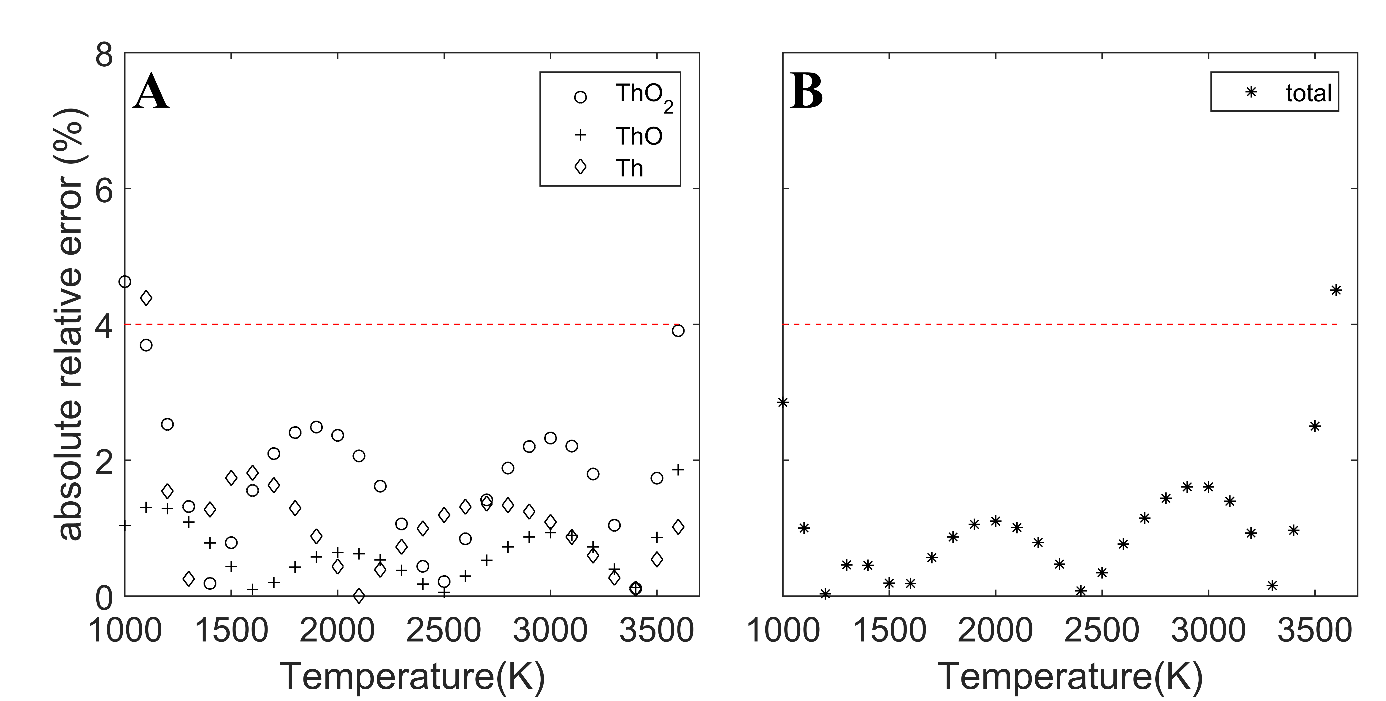


**Figure A1.** Comparison of the partial pressures calculated via the Rand-Markin model and those calculated by FactSage^3^ based on recommended thermodynamic data (at 1 bar ambient pressure). (A) compares the relative errors of the 3 dominant vapour species, while (B) shows the relative error of the total vapour pressure.

The phonon density of states for ThO_2_ have been approximated using a Debye-Einstein approach and are compared to DFT calculation in Figure B1. ThO_2_ has three atoms or ions per molecule and hence 3 acoustic branches (1 longitudinal and 2 transverse) and 6 optical branches (2 longitudinal and 4 transverse). The acoustic branches have been treated via a Debye approach whereby phonons can have a range of frequencies or energies and their respective number follows Bose statistics. Optical modes are approximated by single frequency particles and were represented using the Einstein model. The parameters for this model are summarised in Table B1 and the approach has been described previously^5^. The relative weights can be understood as the fraction of vibration modes characterised by the respective model parameter. The model proposed here is compared to the DFT results for the phonon density of states reported by Wang et al.^6^.

Table B1. – Summary of input parameters for the phonon density of states model.

| Parameter description | units | symbol | value | indicative range | relative  weight | Ref** |
| --- | --- | --- | --- | --- | --- | --- |
| Debye temperatures  (acoustic modes) | K | $\boldsymbol{\vartheta}_{\boldsymbol{TA}\boldsymbol{1}}$ | 170 | 160-216 | 1/9 | ^6,7^ |
|  | K | $\boldsymbol{\vartheta}_{\boldsymbol{TA}\boldsymbol{2}}$ | 260 | - | 1/9 | ^6,7^ |
|  | K | $\boldsymbol{\vartheta}_{\boldsymbol{LA}}$ | 430 | - | 1/9 | ^6,7^ |
| Einstein temperatures  (optical modes) | K | $\mathbf{E}_{\boldsymbol{LO}}$ | 800 | - | 2/9 | ^6,7^ |
|  | K | $\mathbf{E}_{\boldsymbol{TO}\boldsymbol{1}}$ | 630 | - | 2/9 | ^6,7^ |
|  | K | $\mathbf{E}_{\boldsymbol{TO}\boldsymbol{2}}$ | 630 | - | 2/9 | ^6,7^ |

**the Debye and Einstein temperatures have been assigned in this work based on the phonon density of states data reported in the listed references.


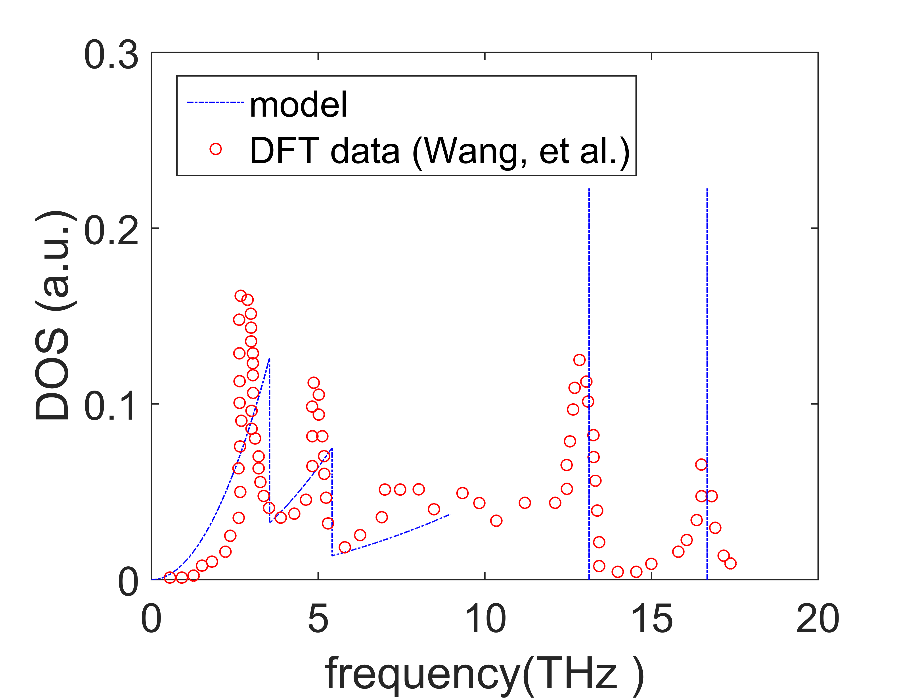


Figure B1. Phonon density of states (PDOS) vs. frequency - comparison between the proposed model for the phonon density of states and the DFT data of Wang et al.^6^

**Supplementary references**

1. Olander, D. R. *Fundamental aspects of nuclear reactor fuel elements*. (1976). doi:10.1016/0022-3115(77)90226-4

2. Rand, M. H. & Markin, T. L. Some thermodynamic aspects of (U,Pu)O2 solid solutions and their use as nuclear fuels. in *Thermodynamics of Nuclear Materials* 637–650 (1967).

3. Bale, C. W., Chartrand, P., Degterov, S. A., Eriksson, G., Hack, K., Mahfoud, R. Ben, Melanqon, J., Pelton, A. D. & Petersen, S. FactSage thermochemical software and databases. *Calphad* **26,** 189–228 (2002).

4. Konings, R. J. M., Benes, O., Kovacs, A., Manara, D., Sedmidubsky, D., Gorokhov, L., Iorish, V. S., Yungman, V., Shenyavskaya, E. & Osina, E. The Thermodynamic Properties of the f-Elements and their Compounds: Part 2. The Lanthanide and Actinide Oxides. *J. Phys. Chem. Ref. Data* **43,** (2014).

5. Pavlov, T., Wenman, M. R., Vlahovic, L., Robba, D., Konings, R. J. M., Uffelen, P. Van & Grimes, R. W. Measurement and interpretation of the thermo-physical properties of UO2 at high temperatures: the viral effect of oxygen defects. *Acta Mater.* (2017).

6. Wang, B. T., Shi, H. L., Li, W. D. & Zhang, P. First-principles study of ground-state properties and high pressure behavior of ThO2. *J. Nucl. Mater.* **399,** 181–188 (2010).

7. Lu, Y., Yang, Y. & Zhang, P. Thermodynamic properties and structural stability of thorium dioxide. *J. Phys. Condens. Matter* **24,** 225801 (2012).
